# Rat Models of Vocal Deficits in Parkinson’s Disease

**DOI:** 10.3390/brainsci11070925

**Published:** 2021-07-13

**Authors:** Maryann N. Krasko, Jesse D. Hoffmeister, Nicole E. Schaen-Heacock, Jacob M. Welsch, Cynthia A. Kelm-Nelson, Michelle R. Ciucci

**Affiliations:** 1 Department of Surgery, University of Wisconsin-Madison, Madison, WI 53792, USA; krasko@surgery.wisc.edu (M.N.K.); hoffmeister@surgery.wisc.edu (J.D.H.); nschaen@wisc.edu (N.E.S.-H.); jmwelsch@wisc.edu (J.M.W.); cakelm@wisc.edu (C.A.K.-N.); 2 Department of Communication Sciences and Disorders, University of Wisconsin-Madison, Madison, WI 53706, USA; 3 Neuroscience Training Program, University of Wisconsin-Madison, Madison, WI 53705, USA

**Keywords:** Parkinson’s disease, rat, ultrasonic vocalization, USV, alpha-synuclein, 6-OHDA, *Pink1*, *DJ1*, exercise, pharmacology, pathology

## Abstract

Parkinson’s disease (PD) is a progressive, degenerative disorder that affects 10 million people worldwide. More than 90% of individuals with PD develop hypokinetic dysarthria, a motor speech disorder that impairs vocal communication and quality of life. Despite the prevalence of vocal deficits in this population, very little is known about the pathological mechanisms underlying this aspect of disease. As such, effective treatment options are limited. Rat models have provided unique insights into the disease-specific mechanisms of vocal deficits in PD. This review summarizes recent studies investigating vocal deficits in 6-hydroxydopamine (6-OHDA), alpha-synuclein overexpression, *DJ1-/-*, and *Pink1-/-* rat models of PD. Model-specific changes to rat ultrasonic vocalization (USV), and the effects of exercise and pharmacologic interventions on USV production in these models are discussed.

## 1. Introduction

Parkinson’s disease (PD) is a progressive, degenerative disorder that affects 10 million people worldwide [1,2]. While the disease is known for hallmark motor signs including a resting tremor, bradykinesia, and rigidity that arise as a result of nigrostriatal dopamine depletion, other signs of disease appear years prior to diagnosis, including changes to voice [3,4,5,6]. More than 90% of individuals with PD develop hypokinetic dysarthria, a motor speech disorder that greatly impairs vocal communication [7,8]. Vocal deficits include decreased loudness, monotone pitch, imprecise articulation, and overall decreased intelligibility [9,10,11,12]. This negatively impacts vocal quality and overall quality of life [12]. Pharmacological treatments for PD typically target dopamine pathways by increasing neurotransmitter levels or as dopamine receptor agonists [13,14]. These treatments, however, are not effective in alleviating voice dysfunction, suggesting pathology for voice differs in important ways from classical limb motor alterations [13,14]. Similarly, surgical treatments, like deep brain stimulation, improve limb motor signs, yet do not improve vocal communication and may in fact worsen deficits [15,16,17,18,19,20,21]. Despite the prevalence of hypokinetic dysarthria in PD, pharmacological and surgical treatment options remain limited. Behavioral therapies continue to be the gold standard in treating voice disorders in this population [22]. While research investigating the efficacy of speech-language interventions for PD-related voice dysfunction has grown, a robust understanding of the underlying biological mechanisms responsible for the onset, progression, and treatment-related improvement in vocal dysfunction is limited. Furthermore, while about 10% of PD cases are familial in nature, a vast majority are deemed idiopathic [23]. There are differences among patients with regard to phenotypic expression of PD, including but not limited to akinetic (freezing), tremor-predominant, young onset, etc. Variability is also noted regarding the presence and severity of signs and symptoms, age of onset, and rate of progression of the disease [24]. This extends to vocal deficits, which often present variably. As such, optimizing treatment remains a universal challenge.

With no yet known etiology and such heterogeneity in the presentation of disease, animal models, including rodent, non-human primate, and non-mammalian, have been used to study different aspects of PD on both behavioral and pathophysiological levels. Rat model systems have allowed for a greater level of experimental control, the ability to study deficits in the prodromal (preclinical) stage of disease, and the means of correlating behavior to neurochemical findings. Specifically, when considering PD-related vocal deficits, neurotoxin, alpha-synuclein, and genetic rodent models have been studied most-extensively and continue to show promise regarding this aspect of disease. The use of animal models has contributed to the PD-voice literature over the last few decades, including characterization of vocal communication in the prodromal stage of disease, assessment of associated neurobiology, especially in extra-dopaminergic pathways, and the development of a training paradigm to study exercise effects on vocal rescue. 

The study of ultrasonic vocalizations (USVs) in rat models of PD has increased understanding of vocal communication changes that occur with PD. Similar to humans, rats are highly social animals, generate sound within the larynx, and produce vocalizations that are semiotic in nature [25,26,27,28,29]. USVs are typically categorized by two call types—22-kilohertz (kHz) alarm calls and 50-kHz calls [30,31,32,33,34,35]. 22-kHz calls occur in response to aversive conditions or in negative affective states and are initiated via activation of the ascending cholinergic system [30,34,36,37,38]. 50-kHz calls occur in response to activity in the mesolimbic dopaminergic system originating in the ventral tegmental area, and are produced in social, nonaggressive, positive affective states [30,31,32,33,34,35]. They represent purposeful affiliative vocalizations, are highly relevant to human communication, and as such, are commonly studied and will be the focus of this review. 50-kHz calls are also more complex, varying by acoustic parameters, such as duration (ms), intensity (dB), bandwidth (Hz), and peak frequency (Hz), as well as non-acoustic parameters, such as complexity (%), call rate (calls/s), latency to call (s), and call type (categorical). There are many different approaches to categorizing call type and categories should correspond to the research question [39,40,41,42,43]. Generally, 50-kHz calls are defined as simple or complex and, depending on the research group, can have sub-categories. Simple calls have constant, non-modulating frequency, and complex calls contain two or more directional changes in frequency of at least 3 kHz each [39,40]. Commonly described complex calls include frequency modulated (FM) calls (frequency changes within a call) and harmonic calls (calls with a fundamental frequency near 30 kHz with a visible harmonic one octave above) [40]. In contrast to human voice, 50-kHz USV production does not involve the vibration of vocal folds [44,45]. USV production shares characteristics with human vocalization including the generation of airflow via buildup of lung pressure, the activation of intrinsic laryngeal muscles, and the modulation of the vocal tract during egressive airflow [28,29]. As such, USVs are used to study vocal sensorimotor control in models of PD. 

**Figure 1:**
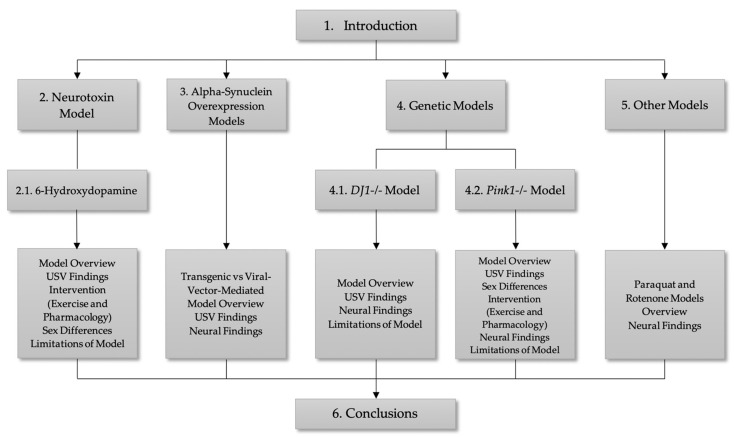
Manuscript overview.

**Figure 2:**
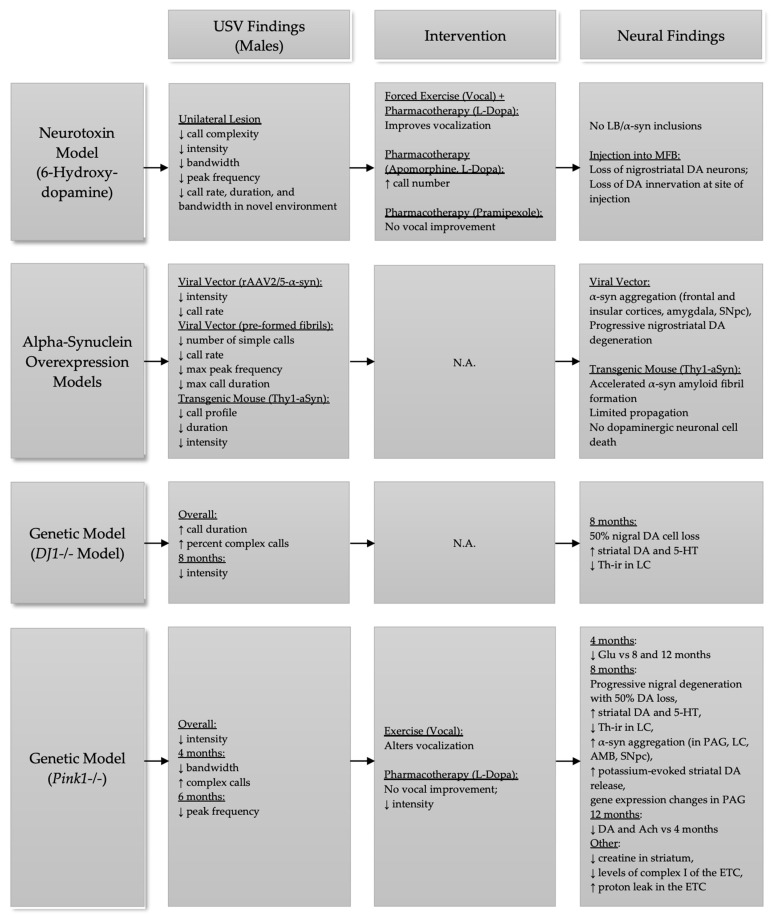
Summary of model-specific USV, intervention, and neural findings in male rats. Ach = acetylcholine, AMB = nucleus ambiguus, α-syn = alpha-synuclein, DA = dopamine, ETC = electron transport chain, Glu = glutamate, LB = Lewy bodies, LC = locus coeruleus, L-Dopa = levodopa, MFB = medial forebrain bundle, PAG = periaqueductal gray, SNpc = substantia nigra pars compacta, Th-ir = tyrosine hydroxylase immunoreactivity, 5-HT = serotonin, ↓ = decrease, ↑ = increase. Underlined text notes category.

The purpose of this focused review is to highlight the changes that occur to 50-kHz vocal communication in various models of PD. This review also expands on model-specific findings regarding targeted exercise and pharmacological interventions for the treatment of vocal deficits in PD, as well as the strengths and weaknesses of these models in studying PD. See Figure 1 for organization of the manuscript and Figure 2 for a summary of pathologic changes across models discussed.

## 2. Neurotoxin Models of PD

### 6-Hydroxydopamine

Oxidopamine, or 6-hydroxydopamine (6-OHDA), is a catecholaminergic neurotoxin classically used to model PD by inducing significant neurodegeneration of the nigrostriatal dopamine system by unilateral or bilateral infusion to the medical forebrain bundle or the striatum [46,47,48,49,50,51,52,53]. The well-established 6-OHDA rat model has been used to study behavioral changes, mechanisms of cell death, and therapies that could potentially improve PD signs [48,54,55,56,57,58]. Deficits in this model are widespread. In addition to affecting limb movements [59,60,61,62,63], unilateral lesions to the medial forebrain bundle or the striatum have been shown to reduce tongue force, lick force, and lick frequency [64,65,66], as well as chewing behaviors [67], suggesting that nigrostriatal dopaminergic systems may contribute, at least in part, to oral sensorimotor dysfunction. 

Nigrostriatal dopamine depletion via unilateral 6-OHDA infusion into the medial forebrain bundle leads to significant changes in USV production. Rat 50-kHz USVs show decreased call intensity, amplitude, and bandwidth [25,68,69]. Additionally, call complexity degrades as a result of the unilateral 6-OHDA lesion. Specifically, there are fewer FM calls and more simple/flat calls without change to the total number of USVs produced (Table 1) [68]. Of all call types (simple, FM, and harmonic), harmonic calls were produced the least frequently; however, this was observed regardless of dopamine depletion [68]. Subsequent work has largely supported these findings, and further showed decreases in call rate, call duration, and bandwidth when tested in a novel cage environment, suggesting that environment can have a significant impact on behavioral outcomes [69]. Observed decreases in complexity and intensity of calls are analogous to hypophonia noted in individuals with PD, thereby demonstrating utility of USVs in assessing phonatory deficits [70]. The effect of time post-lesion on USV production was also studied at acute (72 h) and chronic (4 weeks) timepoints. Results show that after 72 h, call complexity, bandwidth, and intensity of FM calls correlate with striatal dopamine loss. After 4 weeks, bandwidth, intensity of simple calls, and duration of FM calls were correlated with measures of dopamine depletion. Call complexity was less affected at 4 weeks and was only significantly correlated with percent of tyrosine hydroxylase loss [71]. The 6-OHDA model itself does not fully embody the progressive nature of PD. While dopamine loss may play a role in vocal dysfunction, particularly around the time of diagnosis when dopamine has significantly depleted in the substantia nigra pars compacta (SNpc), other systems may be implicated earlier in disease progression that cannot be fully captured with a 6-OHDA model.

Studies have also investigated the potential for exercise and/or pharmacotherapies to rescue deficits induced by the unilateral 6-OHDA lesion. The capacity for forced motor exercise to be neuroprotective against behavioral (motor asymmetry) and neurochemical deficits has yielded mixed results. Forced exercise (casting) implemented prior to induction of toxin-related PD has been shown to be neuroprotective [73], and exercise pre- and post-induced 6-OHDA PD resulted in the prevention of motor deficits and reduced striatal dopamine depletion in adult rats [74,75,76,77]. An intensive 4-week vocal exercise paradigm, in which rats were trained to produce a greater amount of calls with increased complexity and intensity, also rescued decline in call complexity, intensity, and bandwidth compared to no-exercise in the unilateral 6-OHDA model [78]. Lack of physical activity following the 6-OHDA infusion showed exacerbation of behavioral and neurochemical deficits, thereby suggesting dose-dependent relationships between exercise and severity of disease and associated behavioral deficits [79]. 

The effects of vocal exercise, pharmacotherapy, and a combination of vocal exercise and pharmacotherapy (dopamine replacement, e.g., L-Dopa) on USVs produced in familiar and novel environments have also been studied. Vocal exercise post-infusion of 6-OHDA increased maximum peak frequency of USVs; however, this was likely influenced by the testing environment, as the increase was seen in the home cage (compared to novel cage) [69]. Additionally, a combination of vocal exercise and L-Dopa was found to be the most effective treatment approach to increasing peak frequency (as opposed to exercise or L-Dopa alone) and this increase was noted in the novel cage. Findings of this study highlight the benefits of combined interventions, and also suggest the influence that testing environment can have on vocal communication [69].

In addition to L-Dopa, other pharmacotherapies have been trialed for their effects on vocalizations. Drugs that modulate dopamine bioavailability are commonly prescribed to patients with PD in an effort to combat motor deficits throughout disease progression; however, their prolonged use can impact affective state and potentially lead to iatrogenic psychiatric disturbances [80,81,82]. Furthermore, while drugs that target dopamine systems improve motor deficits in humans and animal models of PD [69,83], minimal effect has been observed in rescuing PD-related vocal dysfunction [84]. Simola and colleagues recently observed the effects of three different drugs—apomorphine, L-Dopa, and pramipexole—on USVs in the 6-OHDA rat model. Each drug resulted in different patterns of USV emissions, which is not surprising given their different mechanism of action, with L-Dopa converting into dopamine, pramipexole serving as a dopamine agonist, and apomorphine serving as dopamine receptor agonist. Apomorphine and L-Dopa significantly increased the total number of 50-kHz calls after repeated administration, while pramipexole did not. These findings contrast with previous reports that L-Dopa does not alter the number of calls produced [69]; however, this may be due to differences in the timing of the studies employed between drug administration and USV recording. Overall, results of this study demonstrate that vocal behavior is complex and suggests that the underlying mechanisms driving vocal production are not solely motor in nature [82].

Glial cell-line derived neurotrophic factor (GDNF) has also been trialed in an attempt to establish a neuroprotective therapy and rescue deficits post-lesion. Administration of equine infectious anaemia virus (EIAV) vector coupled with GDNF (EIAV-GDNF) to achieve GDNF expression in the substantia nigra and striatum has been shown to protect dopaminergic neurons in the presence of neurotoxin administration [85]. Furthermore, EIAV-GDNF administration rescued 6-OHDA-induced motor deficits, including rotational asymmetry and spontaneous contralateral motor functions [85,86]. Parkin, the gene product of the PARK2 gene (mutations in which are a major cause of early-onset familial PD), has demonstrated to be neuroprotective in the 6-OHDA model, as well [87]. Overexpressing parkin correlated with improved motor functioning in both cylinder and amphetamine-induced rotation tests [87]. Pharmacotherapies administered prior to or after 6-OHDA infusion have demonstrated utility in rescuing motor deficits; however, further research is necessary to determine the role pharmacotherapies play in rescuing vocalization-related deficits.

Although PD disproportionately affects males, important sex-differences have been identified using animal models of PD. Unilateral 6-OHDA lesioned males and females have shown differences in maintenance of posture, coordination, and initiation of movements [88]. Furthermore, female rats are less susceptible to 6-OHDA than males, specifically, by experiencing significantly less dopaminergic cell loss compared to males after injury [89]. Estrogen has been found to have anti-inflammatory and anti-apoptotic properties on nigrostriatal dopaminergic neurons and, as such, has been suggested to be neuroprotective in females for developing PD [90]. Furthermore, estrogen’s activation of adaptive mechanisms in the nigrostriatal system has been shown to decrease neuronal loss [91] and decrease microglial activation and density, further reducing progression of degeneration [90]. Despite these findings, however, no studies using 6-OHDA to date have focused on the role of estrogen or sex in vocal behavior.

While neurotoxin models can be helpful in investigating behavioral deficits and related neuropathology related to damage in the nigrostriatal pathway, they are not without limitations. Although the 6-OHDA model demonstrates acute neurodegenerative properties, it lacks the ability to induce age-dependent, progressive deficits of PD [92]. Additionally, this model lacks the presence of Lewy bodies, a pathological hallmark in human patients with PD. While unilateral lesions to the medial forebrain bundle are the most common in this model, a disadvantage of this approach is that both A9 (nigrostriatal) and A10 (mesolimbic) cell groups comprise the medial forebrain bundle, and therefore lesioning this site implicates the latter axons [93]. Further, different lesion site selections introduce ambiguity into the model with respect to assessing which site most appropriately approximates clinical PD [93]. Lesion site and unilateral vs. bilateral selections for the model create variation amongst the studied animals; however, 6-OHDA-induced lesions can still provide valuable insight into pathological and related behavioral investigations of PD [93,94]. 

The control of vocalization is complex, involving multiple sensorimotor, cognitive, and limbic brain regions [95,96]. The basal ganglia are certainly implicated in the initiation and modulation of vocalizations. Disrupting nigrostriatal pathways disrupts the quality of vocalization because of altered input to the striatum and consequently the complex circuitry of the basal ganglia and related brain areas. The 6-OHDA lesion to nigrostriatal pathways models one aspect of this complex disease. 

## 3. Alpha-Synuclein Overexpression Models of PD

PD pathology is characterized by the loss of dopaminergic neuronal cells and the formation of misfolded proteins, of which fibrillar alpha-synuclein are the most common, that form Lewy neurites and Lewy bodies in surviving neurons [97]. Mutations include duplications, triplications, or point mutation of the SNCA gene, where alpha-synuclein is the product, which causes autosomal dominant forms of PD [98,99,100,101,102]. Several studies have shown that alpha-synuclein can aggregate and spread, suggesting that it plays a central role in PD progression [103,104,105]. Two common model systems of achieving alpha-synuclein overexpression are genetic modification and viral transduction. Two point mutations of the alpha-synuclein expressing gene (A53T, A30P) have been linked to autosomal-dominant, early onset PD [98,106] and have been shown to accelerate alpha-synuclein amyloid fibril formation [107,108]. Interestingly, transgenic models demonstrate non-motor signs of disease such as olfactory and digestive deficits [109,110]. Additionally, these models show progressive sensorimotor deficits in the absence of dopamine depletion in the striatum, further suggesting alternative mechanisms responsible for these behavioral changes [111]. On the other hand, overexpressing alpha-synuclein using viral vectors models nigrostriatal pathology by injecting within or near the SNpc. In contrast to transgenic models, overexpression via viral vector allows for induction at different timepoints, allows for the targeting of a defined region of the brain, and results in rapid degeneration of nigrostriatal neurons [112]. Furthermore, viral-vector mediated models also show the presence of limb motor deficits [113,114,115,116]. Until recently, vocal deficits were not studied in alpha-synuclein overexpressing models. This is still a largely understudied area, with only two articles discussing vocal deficits in viral-vector-mediated rat models. 

Gombash and colleagues examined USVs of male rats 8 weeks post-administration of 5.9 × 10^13^ rAAV2/5-α-syn injections and compared findings to controls. Duration, bandwidth, intensity, and peak frequency of both simple and FM calls were assessed, as well as call rate and latency to call. Of all acoustic and non-acoustic parameters, only intensity and call rate were found to differ between groups. Specifically, rAAV2/5-α-syn rats produced simple and FM calls with a lower intensity and were found to call at a lower rate compared to controls [117]. Results of this study demonstrated that targeted unilateral nigrostriatal alpha-syn overexpression led to some deficits in USV production. 

The effects of striatal injection of fibrillized mouse alpha-synuclein on USV production have also been studied. Paumier and colleagues demonstrated that rats treated with pre-formed fibrils (PFF) injections produced a lower number of simple calls, had an overall lower call rate, and a significantly reduced maximum peak frequency compared to controls. Furthermore, PFF-treated and recombinant alpha-synuclein rats had shorter maximum call durations compared to their naïve counterparts (Table 2) [118]. However, this study did not directly correlate USV findings to brain neurochemistry. 

Mouse models overexpressing alpha-synuclein [111] have also shown relationships between nigrostriatal alpha-synuclein overexpression and early and progressive decline in behavior. Although not widely studied in the context of vocalization, one additional study characterized vocal deficits in mice overexpressing human wild-type alpha-synuclein under a broad neuronal promoter (Thy1-aSyn) [119]. Grant (2014) found call profile of Thy1-aSyn mice to be significantly different compared to wildtype (WT; healthy) controls. The percent of two-cycle calls and jump down calls was significantly reduced in the Thy1-aSyn model at 2–3 months and 6–7 months, respectively. Furthermore, at 2–3 months, the average duration of calls was significantly decreased (for harmonic, jump down, half cycle, and cycle calls) and at 6–7 months, intensity was significantly reduced in the Thy1-aSyn group. Immunohistochemical findings also revealed alpha-synuclein aggregates in the periaqueductal gray at 5 months in the Thy1-aSyn mice [119]. These deficits coincided with previously reported early sensorimotor deficits, deficits in olfaction, circadian rhythm, and gastrointestinal functioning, and high extracellular striatal dopamine levels [119,120]. Similar to alpha-synuclein overexpressing rat models, mice show early and progressive vocal deficits compared to WTs, suggesting similar underlying mechanisms between both species. Results from these studies indicate that vocal deficits can be induced by alpha-synuclein overexpression, in the absence of dopamine depletion.

## 4. Genetic Models of PD

### 4.1. DJ1-/- Model 

PD is also frequently studied using genetic models. In this model, 2–8 months of age is considered analogous to prodromal to early stage PD in humans. A deletion and missense mutation in the DJ1 (PARK7, Chromosome 1p36) leads to an inherited, autosomal recessive, early onset form of PD [121], that presents with dyskinesia, rigidity, tremors, and later decline in cognitive function. The DJ1 mutation is the second most common identifiable genetic PD etiology after Parkin mutations [122]. Though its role in the pathogenesis of PD is not yet fully understood, DJ1 has been shown to neutralize reactive oxygen species, regulate transcription as well as chaperone, protease, and mitochondrial homeostasis, inhibit alpha-synuclein aggregation, and prevent excessive oxidative stress in the cell [123,124,125]. Thus, deletion of this gene results in a number of behavioral dysfunctions similar to those manifested in human familial PD. 

This review concentrates on the only published paper with regard to vocalization in this rat model. The *DJ1* knockout (*DJ1-/-)* model demonstrates early onset and progressive limb motor, oromotor, and cranial sensorimotor deficits, including decreased limb, tongue/chewing, and vocalization functions. Yang and colleagues (2018) assessed vocalization abilities in the *DJ1-/-* rat model in prodromal to early timepoints of disease (2–8 months of age) and correlated findings to noradrenergic cell loss within the locus coeruleus. Compared to WT controls, *DJ1-/-* rats were found to develop early and progressive ultrasonic vocalization deficits. Specifically, *DJ1-/-* rats produced longer average and maximum calls, and a greater overall percentage of complex calls (Table 3). At 8 months of age, *DJ1-/-* rats showed a lower average intensity of calls, a deficit analogous to the decreased vocal loudness (i.e., hypophonia) PD patients typically experience. Findings also revealed that at 8 months of age, *DJ1-/-* rats demonstrated loss of tyrosine hydroxylase-immunoprotective cells in the locus coeruleus, a brainstem region responsible for the synthesis and regulation of noradrenaline. With widespread connections to the central nervous system, including projections into the prefrontal cortex, striatum, hippocampus, and thalamus, the locus coeruleus has a large impact on PD pathology. Disruptions in the central noradrenergic system are associated with motor and non-motor signs of PD, including vocalization [126]. Tyrosine hydroxylase-positive cells in the locus coeruleus were also found to be negatively correlated with tongue force, suggesting that the greater the loss of neurons within the locus coeruleus, the greater the disruption to oromotor functioning [127]. Whether the loss of these neurons is progressive, however, is still unknown. Overall, noradrenaline has been shown to have widespread implications for PD pathology, including vocalization deficits. 

Other neurotransmitters are also implicated in the *DJ1-/-* rat model [128,129,130]. At 8 months of age, this model shows decreased glutamate release in the striatum and at 4 and 8 months, increased acetylcholine release compared to WTs [130]. Authors of this study, however, speculate that perhaps the difference in neurotransmitter concentrations may have more to do with a change in reuptake rather than release [130]. Studies have also shown that *DJ1-/-* rats are impacted by progressive dopaminergic degeneration in the SNpc as early as 6 months of age, with 50% cell death by the age of 8 months. Interestingly, limb motor deficits including hindlimb grip strength and motor coordination are present before a significant loss of dopaminergic cells. This suggests that the loss of DJ1 may lead to a period of dopamine cell dysfunction that contributes to cognitive impairments in early PD that proceeds cell death [128]. However, the role these systems play in vocal behavior has not yet been studied in this model. 

In contrast to the neurotoxin model, the *DJ1-/-* rat is an early and progressive model, demonstrating unique advantages for the study of vocal deficits in PD. This model also highlights degeneration that occurs outside the classical nigrostriatal dopaminergic system [127], lending to a more robust understanding of the neurobiology underlying voice dysfunction. Despite work by Yang and colleagues, that has characterized vocal behavior in this genetic model, no other studies have looked at *DJ1-/-* vocalizations and little research has focused on the underlying neuropathology. Furthermore, rats used in the majority of studies ranged from 4 to 8 months of age, a period associated with early/prodromal PD. As such, rats in this model may be manifesting their earliest signs of motor and non-motor disruption [121,131], warranting further work that focuses on later time points in PD progression. Similarly, the study of vocalization in this model was completed with males only, underrepresenting sex-specific differences in PD. General exercise has been shown to improve motor function in the *DJ1-/-* model [132], but no exercise paradigm has been evaluated specifically for the improvement of vocalization. 

### 4.2. Pink1-/- Model 

Another gene implicated in early-onset recessive PD cases is *Pink1* [PTEN (phosphatase and tensin homologue)-induced kinase 1; *PARK6*], mutations of which manifest in signs of disease clinically identical to sporadic PD [133,134]. The gene encodes PINK1, a serine/threonine kinase protein that plays a role in autophagic clearance of dysfunctional mitochondria. Deletion or mutation of *Pink1* results in decreased mitochondrial protection against oxidative stress, nigrostriatal dopamine cell death with consequent motor deficits, and non-motor dysfunction, potentially due to extra-dopaminergic breakdown [135,136,137,138]. It is currently the second-most common known cause of autosomal recessive familial PD [139,140]. 

The *Pink1-/-* rat is a well-established model for the study of PD-related behavioral deficits. Rats ages 2–8 months of age represent prodromal and early PD. Similar to *DJ1-/-* rats, *Pink1-/-* rats show early and progressive cranial sensorimotor signs, including vocalization deficits, and associated pathology in the central nervous system and periphery [128]. Of all the models discussed in this review, the *Pink1-/-* rat has been most extensively studied with regard to vocal deficits. As early as 2 months of age (representing prodromal stage), male *Pink1-/-* rats show reductions in vocal intensity [141,142]. This is one of the most commonly disrupted acoustic outcomes in this model and is highly analogous to the reduced vocal loudness almost always seen in patients with PD [8,9,143]. Compared to WTs, *Pink1-/-* rats also demonstrate significantly decreased bandwidth of calls at 2 months of age that progressively worsen to 10 months of age. Furthermore, average peak frequency, which is important for conspecific communication [144], decreases from 2 and 4 months of age to 6 and 8 months of age [141,142,145]. At 6 months of age, peak frequency, maximum intensity, and bandwidth of USVs are significantly decreased in *Pink1-/-* males compared to WT controls. In a playback study of male vocalizations, female rats preferred WT calls and even background noise to *Pink1-/-* calls [146]. By 10 months of age (representing mid-stage), *Pink1-/-* rats continue to demonstrate significant vocal differences compared to WTs. Interestingly, vocal intensity is significantly increased compared to WTs, due to increased motor variability. Motor variability is a hallmark feature of mid-stage PD. Vocalization was also shown to be related to respiratory function. Specifically, elevated minute ventilation, elevated tidal volumes, and lower breathing frequencies were associated with reduced peak frequencies of USVs in *Pink1-/-* rats [145].

Until recently, the study of vocal impairments in progressive genetic animal models of PD has been limited to males. Marquis et al. (2019) were the first to highlight sex-specific differences in the *Pink1-/-* rat by assessing limb sensorimotor, affective state, and vocalization changes in females while also accounting for estrous cycle. [142]. *Pink1-/-* females did not demonstrate a decline in limb motor functioning with disease progression, indicating that perhaps *Pink1-/-* females follow a different time-course for motor dysfunction, compared to males. In terms of affective state, anxiety was heightened in *Pink1-/-* females by 8 months of age. Similarly, by 8 months, vocal loudness for complex (FM) calls decreased in *Pink1-/-* females; however, similar studies in *Pink1-/-* males demonstrated significantly worse vocal deficits across multiple acoustic variables at the 8-month and earlier timepoints (Table 4), suggesting that there may be a sex-specific difference in vocal degradation, with female vocalization breakdown occurring at a slower rate. 

A number of studies have attempted to restore vocalization in *Pink1*-/- rats through behavioral and pharmacologic interventions. Similar to the lack of improvement in vocal communication in humans who are prescribed medications, levodopa has a minimal impact on rat vocalization, and can even further reduce vocal intensity [147]. This provides support to the evolving hypothesis that neuropathology associated with vocalization deficits in PD is at least partially extra-dopaminergic. In contrast, behavioral intervention (vocal exercise) rescues some acoustic aspects of USVs, however, these changes are most effective in early stages of disease progression [148], and do not necessarily “normalize” to WT-like calls [149]. 

Among the most-important advantages of the *Pink1-/-* rat model of PD is the ability to study both neural tissue and peripheral disease pathology (cranial muscles and nerves) and to correlate findings to vocalization behavior. While findings of nigrostriatal dopamine depletion are inconsistent in this model [150], deficits in other brain regions and neurotransmitter systems are common and reproducible [138]. The number of tyrosine hydroxylase immunoreactive cell bodies in the locus coeruleus is reduced in *Pink1-/-* rats, and these cell counts are correlated with vocalization intensity deficits [141]. In *Pink1-/-* rats, protein concentration of norepinephrine in the locus coeruleus is reduced and catechol-o-methyltransferase gene expression in the locus coeruleus is increased. Furthermore, catechol-o-methyltransferase mRNA expression is associated with a percentage of complex calls [147]. Increased levels of striatal serotonin (5-HT) have been reported in 8-month-old *Pink1-/-* rats compared to age-matched controls [128,151], similar to findings of prior studies in other models of PD [152,153]. However, other studies have not found significant differences in striatal [130] or dorsal raphe 5-HT levels between *Pink1-/-* rats and WTs [145]. Further exploration is warranted. 

*Pink1-/-* rats show significant differences in vocal brain regions and in laryngeal and tongue muscles. For example, 8-month old *Pink1-/-* rats have increased alpha-synuclein in the periaqueductal gray yet show no significant mRNA expression of alpha-synuclein compared to WT controls. Instead, *Pink1-/-* rats demonstrate decreased expression of *Atp13a2*, a lysosomal P-type transport ATPase, in the periaqueductal gray [154], suggesting a possible mechanism for alpha-synuclein aggregation. Furthermore, Glass and colleagues recently demonstrated that ex-vivo thyroarytenoid muscles of *Pink1*-/- rats produce decreased force levels in response to 1-Hz and 20-Hz stimulations and show significantly different proportions of myosin heavy chain isoforms relative to WTs, namely an increase in 2L and a reduction in 2X isoforms [155]; work relating this to progression of vocal deficits is ongoing. 

Finally, Kelm-Nelson and Gammie recently used high-throughput RNA sequencing to identify differences in gene expression in the periaqueductal gray (a vocal modulatory region) in male and female *Pink1-/-* rats compared to WT controls [156]. Subsequent weighted gene co-expression network analysis identified correlations between relevant gene modules and vocalization in female *Pink1-/-* and WT rats. Differentially expressed genes for both male and female rats mapped to human PD datasets, suggesting that the rat model closely aligns to human PD. This work highlights the potential for the *Pink1*-/- rat to be used to identify targeted pharmacologic interventions for vocal deficits in PD. 

## 5. Other Models

PD etiology remains largely unknown. With such a high number of cases being deemed idiopathic, other models have been developed to study how environment contributes to pathogenesis. Pesticides and herbicides play a role in the development of PD. Paraquat, an herbicide commonly used in agriculture, has been linked to PD via experimental work in rodents. Exposure leads to alpha-synuclein upregulation, increased alpha-synuclein aggregation, microglial activation, oxidative stress, dose-dependent loss of TH-positive striatal fibers and SNpc neurons, and reduced motor activity [157,158,159]. As reviewed by Nandipati and Litvan (2016), several studies have also explored the relationship between paraquat and PD in humans; while research is not entirely consistent, most findings show that exposure to paraquat is linked to PD [160].

Another environmental toxin used to study PD is rotenone—a lipophilic insecticide and herbicide that crosses the blood–brain barrier and serves as a complex I inhibitor in cellular respiration. Experimental animals exposed to rotenone show robust signs of PD, including alpha-synuclein aggregation in the brain and periphery, inflammation and activation of microglia, mitochondrial dysfunction, oxidative stress, and behavioral deficits, including motor, postural, and gastrointestinal dysfunction [161,162,163]. While rotenone has a rather selective toxicity toward dopaminergic cells [164,165], rotenone also causes neurodegeneration of striatal serotonergic and cholinergic cells, as well as noradrenergic cells in the locus coeruleus. Additionally, administration of rotenone results in impairment of locomotor activity, providing evidence that other neurotransmitters in addition to dopamine may be implicated in behavioral changes [165].

Although vocalizations have not been assayed in models of environmental toxins, given the number of parallels observed in these models to idiopathic PD, studying vocalization could provide critical insights into the mechanisms responsible for vocal decline in idiopathic PD. Findings could also contribute to earlier disease identification, and guide the development of new behavioral and pharmacological interventions for PD-related voice disorders. 

## 6. Conclusions

Rat models have contributed to our understanding of PD. While hallmark motor deficits are relatively well-understood, certain signs of PD, including vocal deficits, remain poorly understood due to their prodromal onset and complex pathology. As such, multiple complementary models are necessary to provide insights into the progression and pathophysiological underpinnings of communication deficits. In this paper, we discussed neurotoxin, alpha-synuclein mutation, and genetic rat models that have recently been used to interrogate mechanisms of vocal communication impairment in PD. We reviewed model-specific changes to USV production and associated neurochemistry, and reviewed the role of exercise and pharmacological interventions in vocal rescue. Each of the different models of PD have unique advantages and limitations. Neurotoxin models such as 6-OHDA are useful for the study of mid- to late-stage PD associated with nigrostriatal dopamine depletion, and demonstrate widespread deficits; however, this model shows minimal alpha-synuclein aggregation and does not account for the progressive nature of the disease. In contrast, genetic models like *DJ1-/-* and *Pink1-/-* allow for the study of disease progression, as well as the study of intervention at early, prodromal, and later timepoints. However, genetic mutations make up only a small subset of PD cases and may not capture the subtle differences associated with the pathogenesis of other forms of PD. Paraquat and rotenone models display many signs of PD; however, the effect of these environmental toxins on vocalization is still unknown. Although no one model fully captures the complexity of PD, these models serve as a valuable tool for expanding our understanding of the disease and translating findings to human populations to advance identification and treatment.

## Figures and Tables

**Table 1:** Summary of main effects of dopamine condition and call type on USV production.

Publication	Sex	Dopamine Conditions (Independent Variable)	Call Type (Independent Variable)	USV (Dependent Variables)	Effect	Finding
Ciucci et al., 2007 [72]	M	6-OHDA, Haloperidol, Control	n.a.	bandwidth	main effect of DA condition	6-OHDA bandwidth ↓
				number of calls	n.s.	
Ciucci et al., 2008 [25]	M	6-OHDA, Haloperidol, Control	n.a.	peak amplitude	significant	6-OHDA peak amplitude ↓
Ciucci et al., 2009 [68]	M	6-OHDA, Haloperidol, Control	Simple, FM	percent simple calls	significant	Percent of simple calls > harmonic
				percent FM calls	significant	Percent FM calls > harmonic
				percent call type	significant	Simple was most frequent in 6-OHDA
						FM was most frequent in haloperidol and controls
				total number of calls	n.s.	
				duration	n.s.	
				bandwidth	main effect of DA condition	6-OHDA bandwidth ↓
				maximum frequency	main effect of call type	Maximum frequency in FM > simple
				maximum intensity	main effect of DA condition	6-OHDA intensity ↓
					main effect of call type	Maximum intensity in FM > simple

DA = dopamine, FM = frequency modulated, M = male, n.a. = not applicable, n.s. = not significant, USV = ultrasonic vocalization, ↓ = decrease.

**Table 2:** Summary of effects of alpha-synuclein treatment on USV production.

Publication	Sex	Treatment Group	USV (Dependent Variables)	Effect	Finding
Gombash et al., 2013 [117]	M	rAAV2/5-α-syn, controls	call type	n.s.	
			duration	n.s.	
			bandwidth	n.s.	
			intensity	significant	rAAV2/5-α-syn intensity ↓
			peak frequency	n.s.	
			call rate	significant	rAAV2/5-α-syn call rate ↓
			latency to call	n.s.	
Paumier et al., 2015 [118]	M	recombinant α-syn, α-syn PFF, controls	number of calls	treatment x call type interaction effect	α-syn PFF number of simple calls ↓
			call rate	main effect of treatment	α-syn PFF call rate ↓
			duration	main effect of treatment	recombinant α-syn and α-syn PFF duration ↓
			peak frequency	main effect of treatment	α-syn PFF peak frequency ↓
			intensity	n.s.	
			latency to call	n.s.	

α-syn = alpha-synuclein, M = male, n.s. = not significant, USV = ultrasonic vocalization, ↓ = decrease.

**Table 3:** Summary of interaction effects and main effects of genotype and age on USV production between *DJ1-/-* rats and WT controls.

Publication	Sex	Genotypes (Independent Variable)	Ages (mo) (Independent Variable)	USV (Dependent Variables)	Effect	Finding
Yang et al., 2018 [127]	M	*DJ1-/-*, WT	2, 4, 6, 8	percent complex calls	main effect of genotype	*DJ1-/-* percent complex calls ↑
					main effect of age	Percent complex calls at 6 and 8 mo > 2 mo;
						Percent complex calls at 6 and 8 mo > 4 mo
				maximum duration	main effect of genotype	*DJ1-/-* maximum duration ↑ (longer)
					main effect of age	Maximum duration at 4 mo > 2 mo
				maximum bandwidth	main effect of age	Maximum bandwidth at 4 mo > 2 mo
				maximum intensity	main effect of age	Maximum intensity at 4, 6, and 8 mo > 2 mo
				maximum peak frequency	n.s.	
				average duration	main effect of genotype	*DJ1-/-* average duration ↑
				average bandwidth	main effect of age	Average bandwidth at 4 mo > 2 mo
				average intensity	genotype x age interaction	At 8, *DJ1-/-* average intensity ↓
				average peak frequency	main effect of age	Average peak frequency at 8 mo < 2 mo

M = male, mo = months, n.s. = not significant, USV = ultrasonic vocalization, WT = wildtype, ↓ = decrease, ↑ = increase.

**Table 4:** Summary of interaction effects and main effects of genotype and age on USV production between *Pink1-/-* rats and WT controls.

Publication	Sex	Genotypes (Independent Variable)	Ages (mo) (Independent Variable)	USV (Dependent Variables)	Effect	Finding
Grant et al., 2015 [141]	M	*Pink1-/-*, *Pink1+/-,* WT	2, 4, 6, 8	average intensity of FM calls	main effect of genotype	*Pink1-/-* average intensity ↓ vs. WT and *Pink1+/-*
					main effect of age	Average intensity at 4 mo > 2 mo
				average bandwidth of FM calls	genotype x age interaction	At 4 and 6 mo, *Pink1-/-* average bandwidth ↓ vs. WT;
						*Pink1-/-* average bandwidth at 4, 6, and 8 mo < 2 mo
				average peak frequency of FM calls	genotype x age interaction	At 6 and 8 mo, *Pink1-/-* average peak frequency ↓ vs. *Pink1+/-*;
						*Pink1-/-* average peak frequency at 6 and 8 mo < 2 and 4 mo;
						At 6 and 8 mo, *Pink1+/-* average peak frequency ↑ vs. WT
				average duration of FM calls	main effect of age	Average duration at 4, 6, and 8 mo > 2 mo
				percent complex calls	genotype x age interaction	All rats produced more complex calls over time;
						*Pink1-/-* complex calls at 4, 6, and 8 mo > 2 mo;
						WT complex calls at 8 mo > 4 mo;
						*Pink1+/-* and WT complex calls at 6 and 8 mo > 2 and 4 mo
Johnson et al., 2020 [145]	M	*Pink1-/-*, WT	10	intensity	main effect of genotype	*Pink1-/-* intensity ↑
				peak frequency	main effect of genotype	*Pink1-/-* peak frequency ↓
				average duration of FM calls	n.s.	
				average bandwidth	n.s.	
Marquis et al., 2020 [142]	F	*Pink1-/-*, WT	2, 4, 6, 8	number of calls	genotype x age interaction	At 2 mo, *Pink1-/-* number of calls ↑;
						*Pink1-/-* and WT number of calls at 2 mo > 4, 6, and 8 mo
				percent complex calls	main effect of age	All rats produced ↓ percent complex calls over time;
						Significant differences between 2-4, 2-8, 4-6, 4-8, and 6-8 mo
				duration of simple calls	main effect of age	Duration of simple calls significantly different at 4 mo vs. 2, 6, 8 mo
				duration of complex calls	main effect of age	Duration of FM calls at 8 mo > 2 and 4 mo
				bandwidth of simple calls	main effect of age	Bandwidth of simple calls at 4 mo < 2, 6, 8 mo
				average intensity of FM calls	main effect of genotype	*Pink1-/-* average intensity of FM calls ↓

F = female, FM = frequency modulated, M = male, mo = months, n.s. = not significant, USV = ultrasonic vocalization, WT = wildtype, ↓ = decrease, ↑ = increase.
